# Supplementary material for: Effects of Zearalenone on Apoptosis and Copper Accumulation of Goat Granulosa Cells In Vitro
Source: Biology (Basel). 2023 Jan 9;12(1):100. doi: 10.3390/biology12010100 (PMC9856194; doi:10.3390/biology12010100)
Supplement: Supplementary file 1 [file biology-12-00100-s001.zip › biology-2104355-supplementary proofdone.pdf]

Supplementary Materials

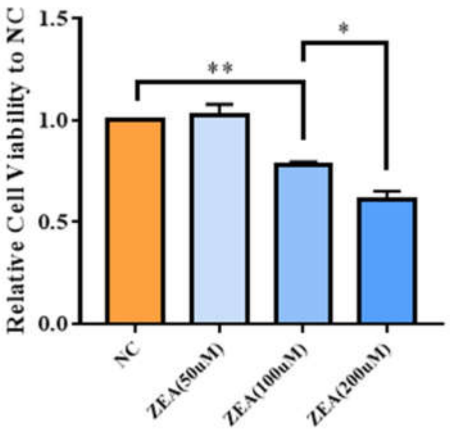

Figure S1. CCK-8 results of GCs treated with different concentration of ZEA. GCs were treated with 0, 50, 100 and 200 μmol/L ZEA and cell viability was detected by the CCK-8 Kit. All the experiments were performed in triplicates, each value is the mean ± SEM. * $p < 0.05$, ** $p < 0.01$.

Table S1. Details of primer sequences, expected product sizes and Genbank accession numbers of genes used for qRT-PCR.

Items	Primer sequence (5′-3′)	Genebank No.	Fragment size (bp)
PCNA	F: TGGCTCCCAAGATCGAGGAT<br>R: TAAAACTGCATTTAGAGTCAAGACC	NM_002592.2	381
CASP3	F: TGAAACATGCCGCCTTCCTA<br>R: AGTGGCATACCCACATGACTG	XM_018041755.1	242
CASP9	F: CCAGATGCCGTGTCTAGTCTG<br>R: ACAGTAAGGTAGGGTGAGGGG	XM_005690814.3	296
STAR	F: GGTCCCCGAGACTTTGTGAG<br>R: AATCCACTTGGGTCTGCGAG	XM_013975437.2	262
CYP11A1	F: CACTTTCGCCACATCGAGAAC<br>R: AGGCTCCTGACTTCTTAAACAGG	NM_001287574.1	217
3B-HSD	F: AGACCAGAAGTTCGGGAGGAA<br>R: TCTCCCTGTAGGAGTTGGGC	XM_013962473.2	292
CYP19A1	F: TGGTGTCCGAAGTTGTGCCTATTG<br>R: AAGGTCGAACAGCTTTCCAGAGTG	XM_013967046.2	393
CYP17A1	F: AACGCCATAGCAAGGAACGA<br>R: TCTGTCACGCTGTGTTGTGT	NM_001255003.3	263
BAX	F: GCATCCACCAAGAAGCTGAG<br>R: CCGCCACTCGGAAAAAGAC	XM_002701934.1	130
BCL2	F: ATGTGTGTGGAGAGCGTCA<br>R: AGAGACAGCCAGGAGAAATC	NM_001166486.1	182
CCND1	F: AGACCCTCGCTTGTGCTTAC<br>R: AACGTGCCGGTTACATGTCT	XM_018043271.1	117
CDK4	F: CCTTCATGCCAACTGCATCG<br>R: GGCAGTCCGATCAGGTCAAA	XM_005680266.3	309

CDK6	F: GATGGGTTTCCACCAGGGAG<br>R: CTTTGTAACCCGATGCTGCG	XM_018047424.1	203
TP53	F: TTCAAAAGTCCAGAGCCACCA<br>R: GGACATTCATCCAGCCAGGT	XM_005693530.3	316
CAT	F: CATTACCAGATACTCCAAGGCGAAGG<br>R: TGGCTATGGATAAAGGACGGAAACAG	XM_005690077.3	234
SOD2	F: CGGCCTACGTGAACAACCTCAAC<br>R: GGACACCAACAGATACAGCAGTCAG	XM_018053428.1	261
GSH-px	F: GGTCCAACCGTCACTCAACT<br>R: ACCATTCCGGTTTCTCGCTT	XM_011494791.2	389
SLC31A1	F: CACCATCACCCGACCACATCATC<br>R: AGCCATTTCTCCAGCCGTATTGATC	XM_005684318.3	138
MT2A	F: CGGCTCCTGCAAATGCAAAGATTG<br>R: TCAGGCACAGCAACTGCACTTG	JK747640.1	139
ATP7A	F: GAAACGGGTAGCAATGGTAGGAGAC<br>R: CGTCTGTGCCTGTGCCAATAGC	XM_005700602.3	92
ATP7B	F: TGACAACAATGGCTACGAGGATGAC<br>R: CGATGGACTTGACACACGACTGG	XM_018056698.1	110
HSP70	F: CGACGACGGCATCTTCAAGGTG<br>R: TGTTCTGGCTGATGTCCTTCTTGTG	NM_001285703.1	134
GAPDH	F: CCGTTCGACAGATAGCCGTAA<br>R: CCGTTCGACAGATAGCCGTAA	XM_005680968.3	296
LIPT1	F: TGTGGTAATTGGTCGGCATCAGAAC<br>R: CTCCGAGCCAGTTTCACACCTTC	XM_005686281.3	84
LIAS	F: CAGTGTGTGAGGAAGCTCGATGTC<br>R: CATGATTGTGGCTGTGGCGGTAG	XM_005681520.3	83
DLD	F: GCCACAGGTTCAGAAGTCACTCC<br>R: TGCCAAACTGAGCCCAATTCTACAC	XM_005679140.3	155
DLAT	F: ACCACCAGCACCACCACCTATC<br>R: GGCTAACGAACACTCTTCCCTTTGG	XM_018059784.1	131
PDHA1	F: GCAGCCAGCACTGACTACTACAAG<br>R: ACGGTAACTCACTCCAGGATCACTC	XM_018044485.1	198
PDHB	F: CTGAGATGGGTTTTGCTGGAATTGC<br>R: GAAGACTATGGGCACAGACTGAAGG	XM_005695783.2	170

Table S2. Details of antibodies.

Antibodies name	Cat NO.	Source	Dilutions used in IHC	Dilutions used in WB	Dilutions used in IF
Anti-PCNA antibody	Ab18197	Abcam	–	1:500	–
BAX Rabbit Polyclonal antibody	50599-2-Ig	Proteintech	–	1:2000	–
Bcl-2(D17C4) Rabbit mAb	3498	Cell Signaling Technology	–	1:500	–
Caspase 3 Rabbit Poly-clonal antibody	19677-1-AP	Abcam	–	1:1000	–
CYP11A1 polyclonal antibody	bs-10099R	Bioss	–	1:1000	–
Caspase 9 Rabbit Poly-clonal antibody	10380-1-AP	Abcam	–	1:1000	–

Cytochrome P450 19A1 antibody	DF6884	LTD	−	1:2000	−
CDK6 Polyclonal Antibody	14052-1-AP	Proteintech	−	1:2000	−
Cyclin D1 Monoclonal Antibody	60186-1-IG	Proteintech	−	1:2000	−
CDK4 Polyclonal antibody	11026-1-AP	Proteintech	−	1:2000	−
SOD2 Polyclonal antibody	24127-1-AP	ProteinTech	−	1:2000	−
Catalase Monoclonal antibody	66765-1-Ig	ProteinTech	−	1:2000	−
P53 Monoclonal antibody	60283-2-Ig	ProteinTech	−	1:1000	−
SLC31A1 Rabbit pAb	A10109	ABclonal	−	1:500	−
MT2A Rabbit pAb	A2018	ABclonal	−	1:500	−
ATP7A Rabbit pAb	A8399	ABclonal	−	1:500	−
HSP70/HSPA1 Rabbit pAb	A12948	ABclonal	−	1:1000	−
ATP7B Ab	AF0410	Affinity	−	1:1000	−
Anti-beta Actin antibody	ab8227	Abcam	−	1:1000	−
Alpha Tubulin Monoclonal Antibody	66031-1-Ig	Proteintech	−	1:1000	−
GAPDH Mouse Monoclonal antibody	60004-1-Ig	ProteinTech	−	1:8000	−
HRP-conjugated Affinipure Goat Anti-Rabbit IgG(H+L)	SA00001-2	ProteinTech	−	1:5000	−
HRP-conjugated Affinipure Goat Anti-Mouse IgG(H+L)	SA00001-1	ProteinTech	−	1:5000	−

IHC: immunohistochemistry, WB: Western blot, IF: immunocytochemistry, (−): absent.
