# Supplementary figures and images for: Effects of Zearalenone on Apoptosis and Copper Accumulation of Goat Granulosa Cells In Vitro
Source: Biology (Basel). 2023 Jan 9;12(1):100. doi: 10.3390/biology12010100 (PMC9856194; doi:10.3390/biology12010100)

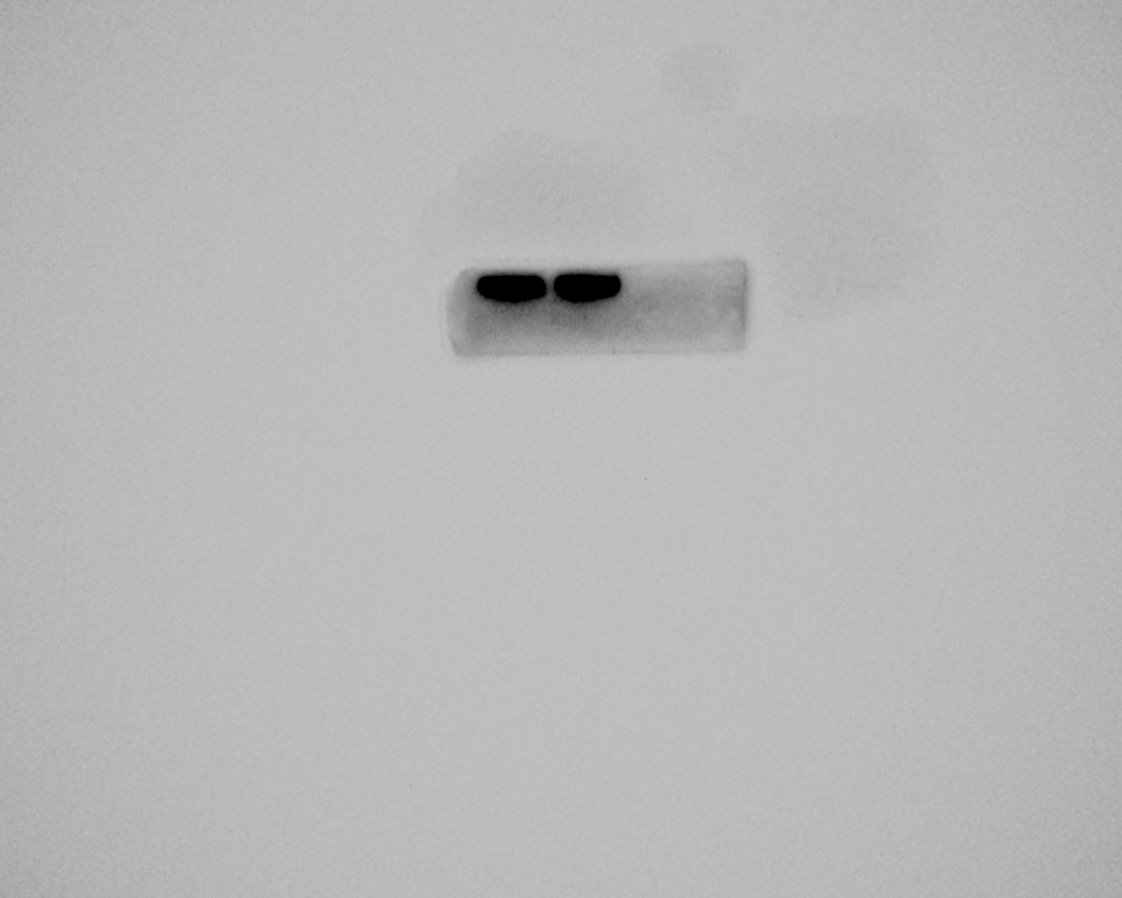

Supplement: Supplementary file 1 [file biology-12-00100-s001.zip › actb.tif]

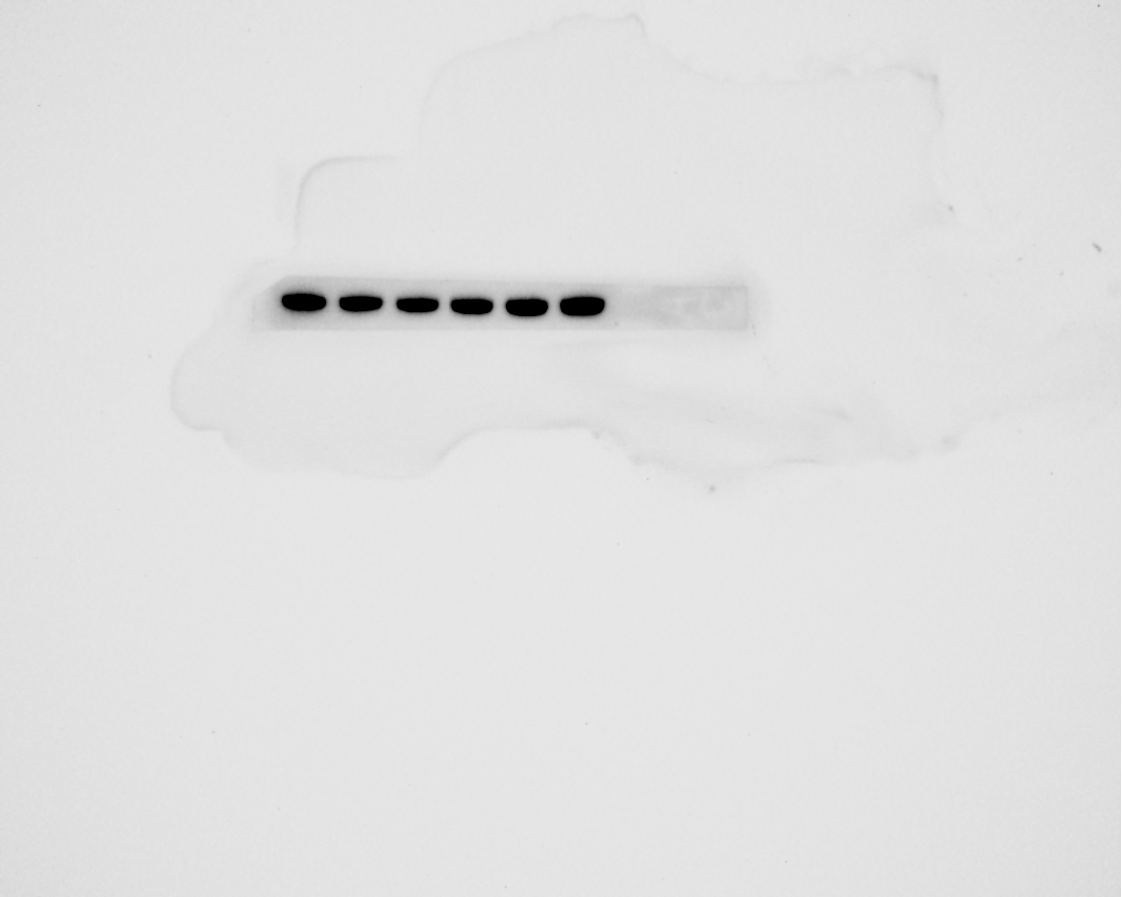

Supplement: Supplementary file 1 [file biology-12-00100-s001.zip › actb2.tif]

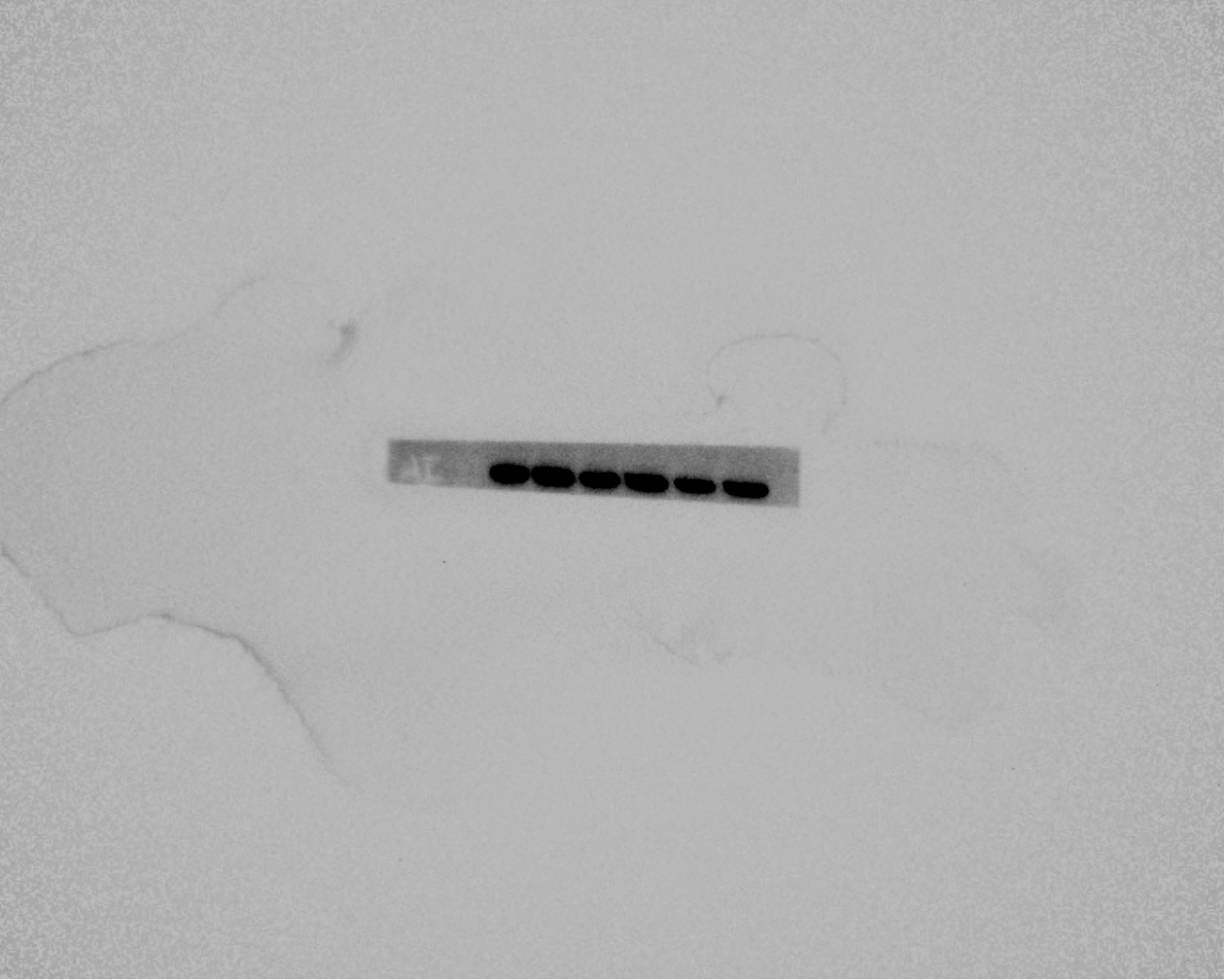

Supplement: Supplementary file 1 [file biology-12-00100-s001.zip › actb3.tif]

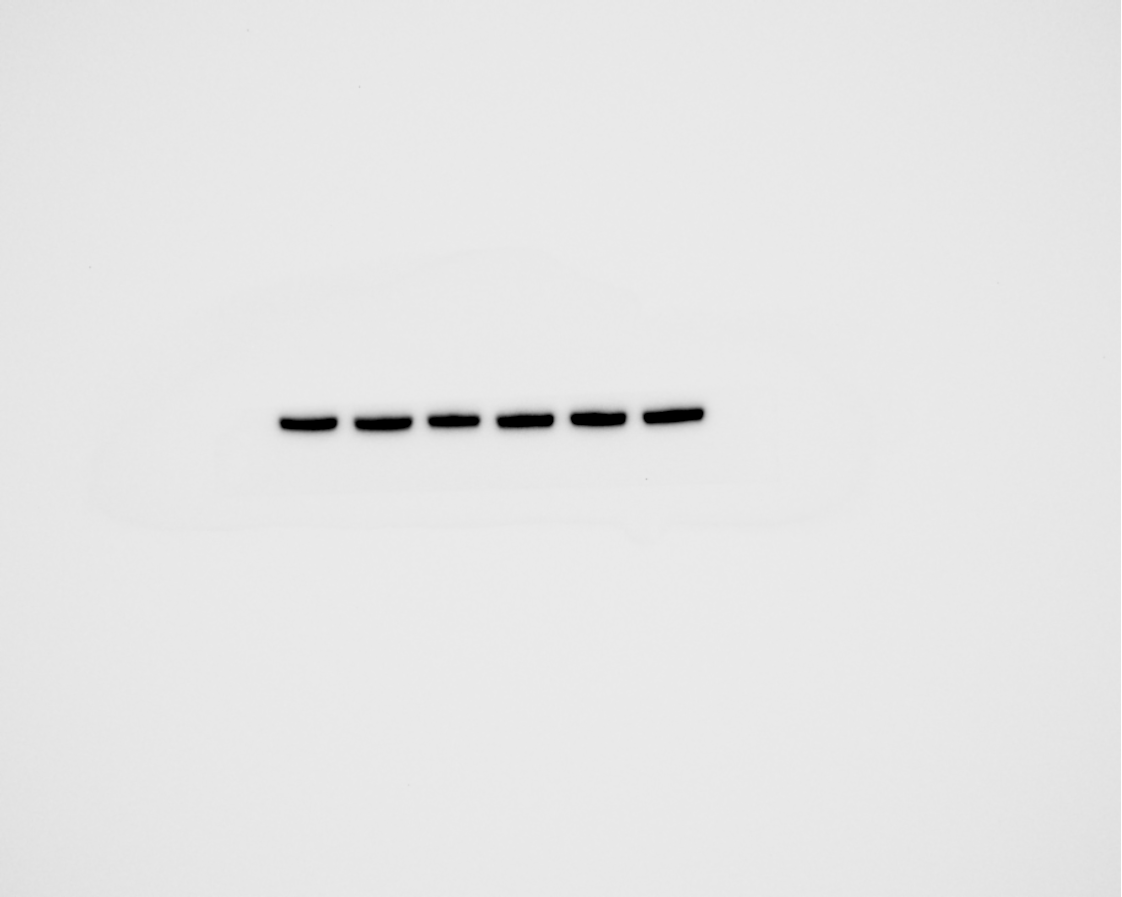

Supplement: Supplementary file 1 [file biology-12-00100-s001.zip › actb4.tif]

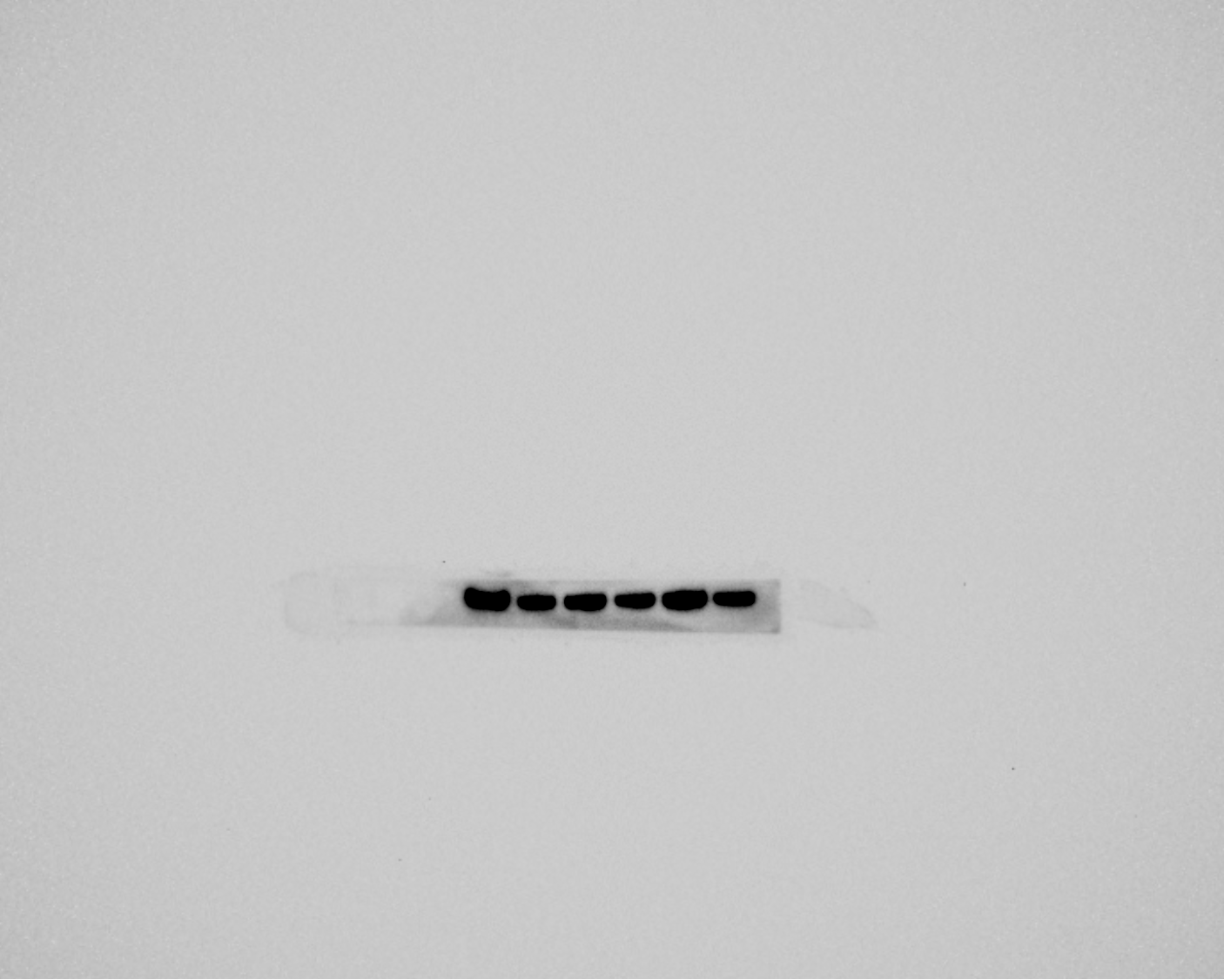

Supplement: Supplementary file 1 [file biology-12-00100-s001.zip › actb7.tif]

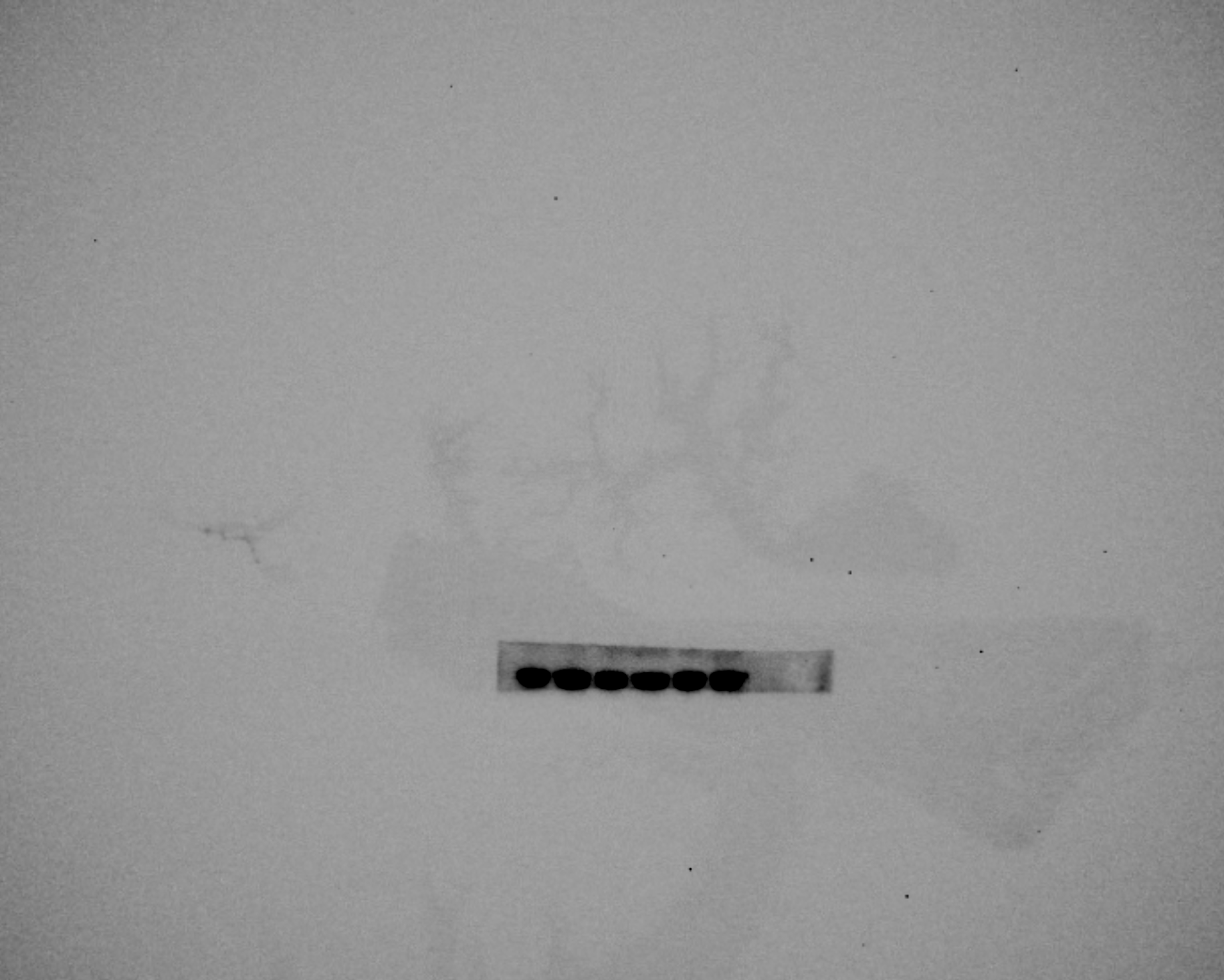

Supplement: Supplementary file 1 [file biology-12-00100-s001.zip › actb8.tif]

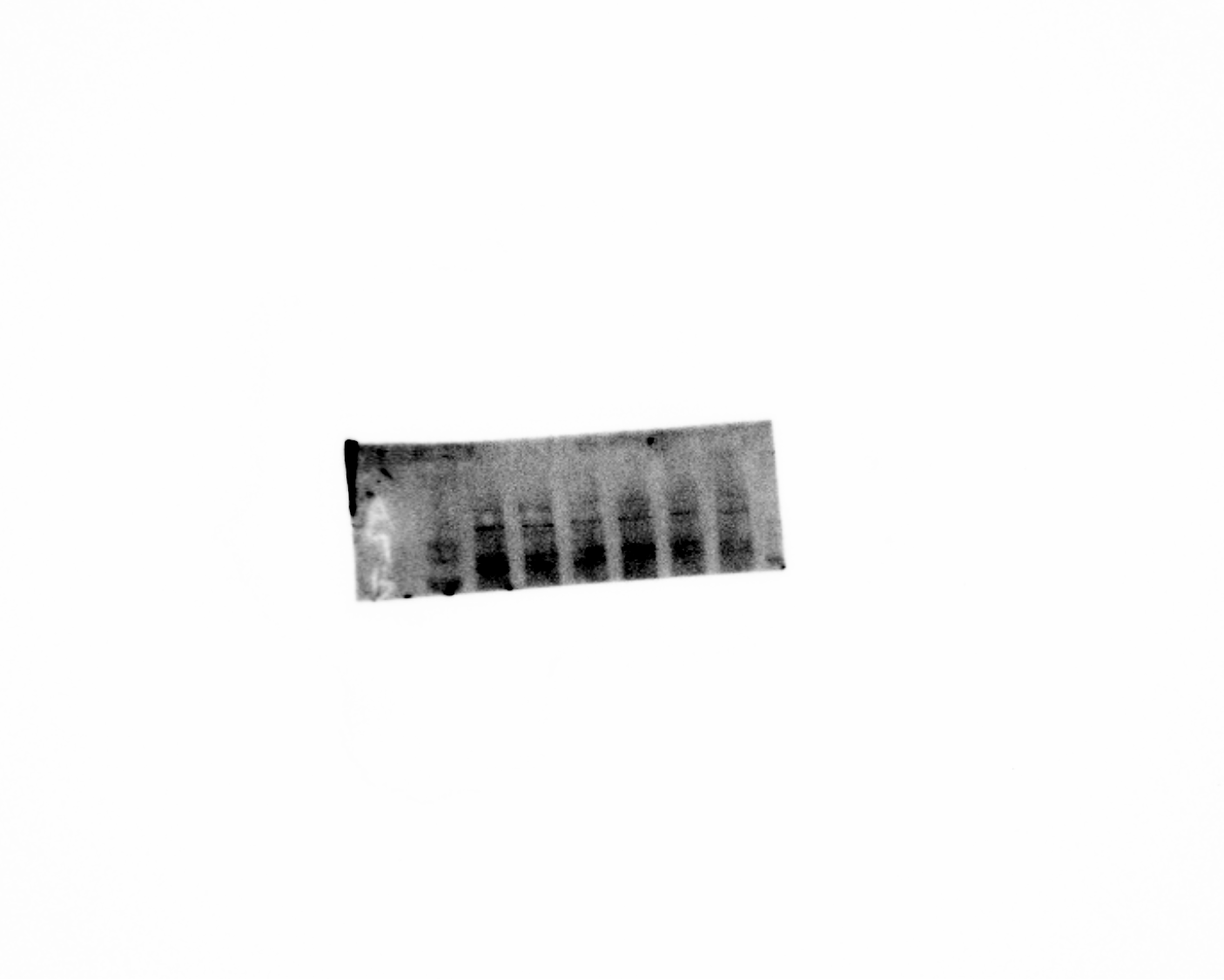

Supplement: Supplementary file 1 [file biology-12-00100-s001.zip › atb7b.tif]

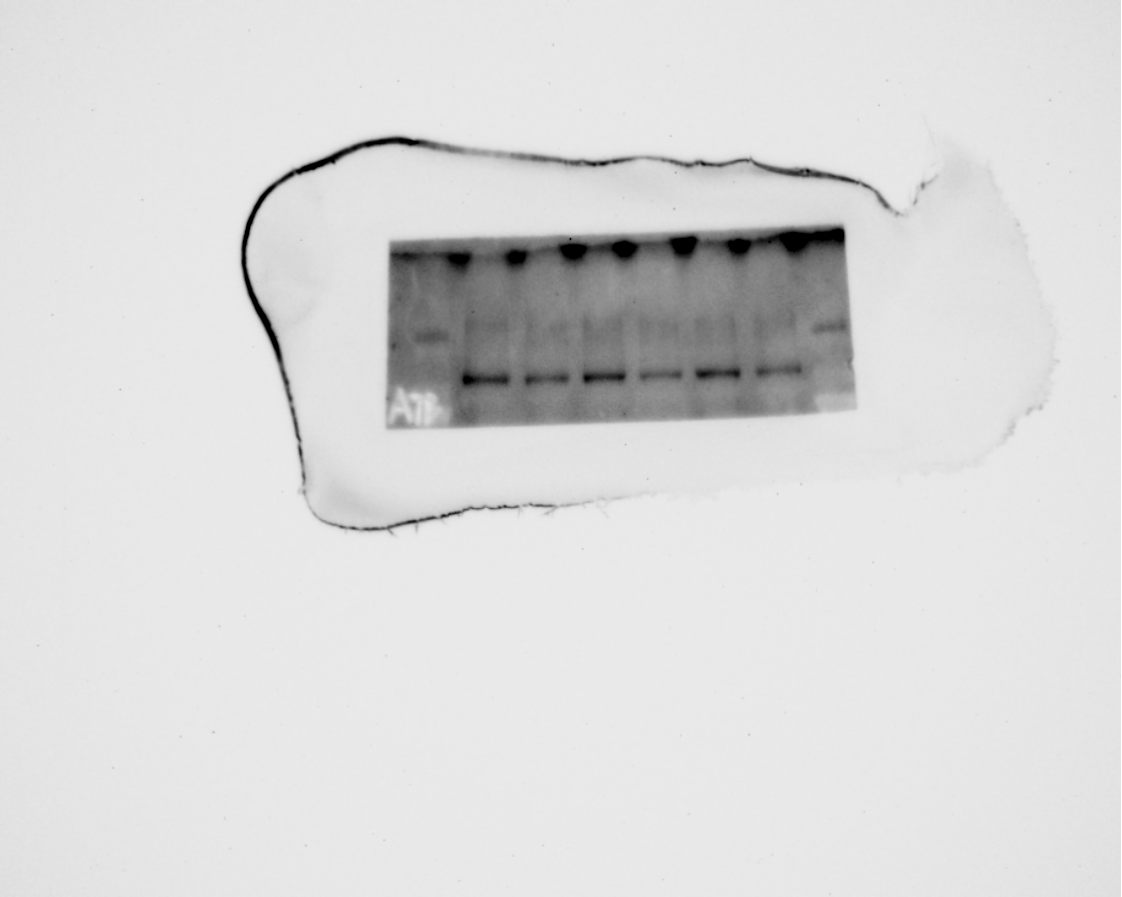

Supplement: Supplementary file 1 [file biology-12-00100-s001.zip › atp7a.tif]

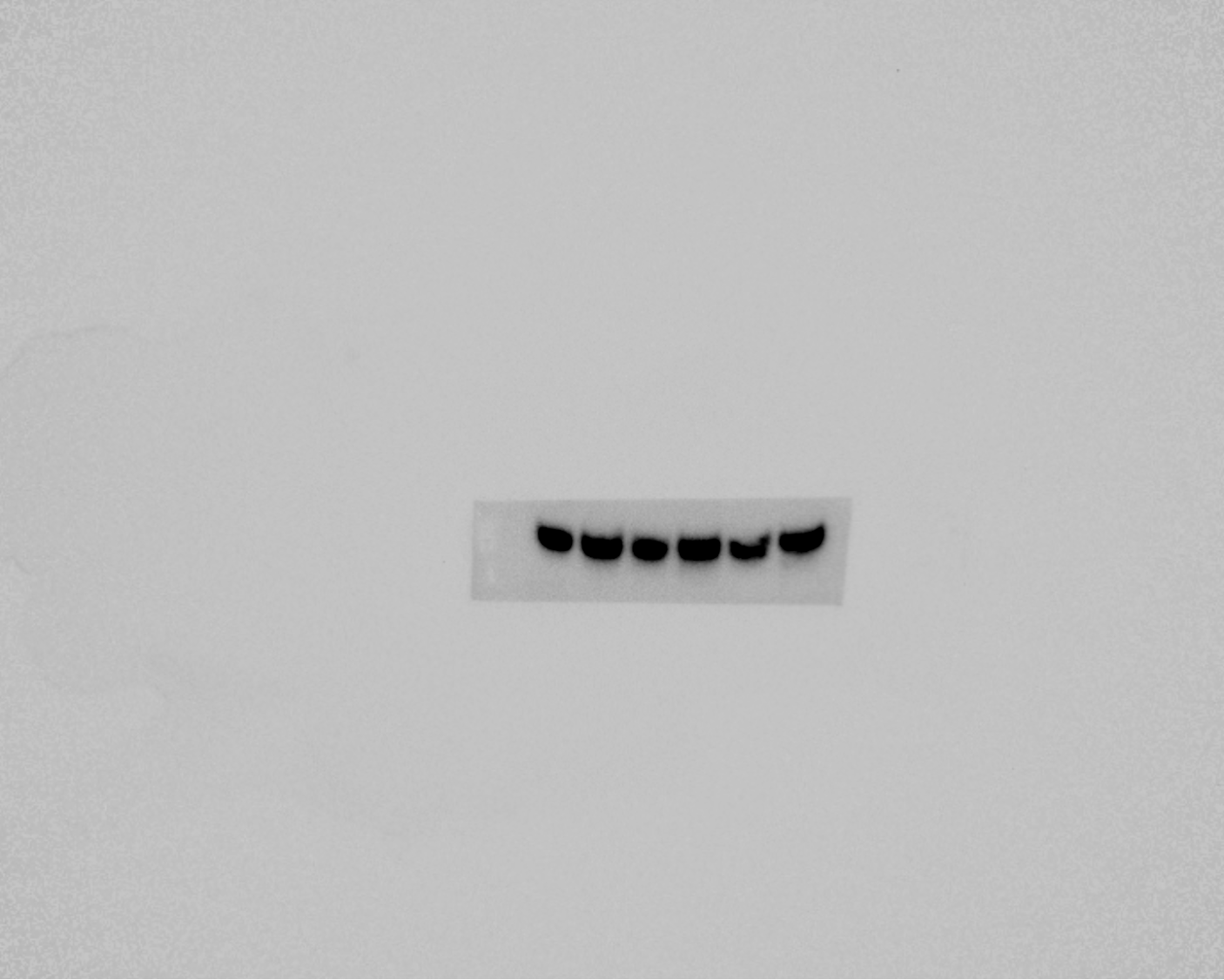

Supplement: Supplementary file 1 [file biology-12-00100-s001.zip › bax.tif]

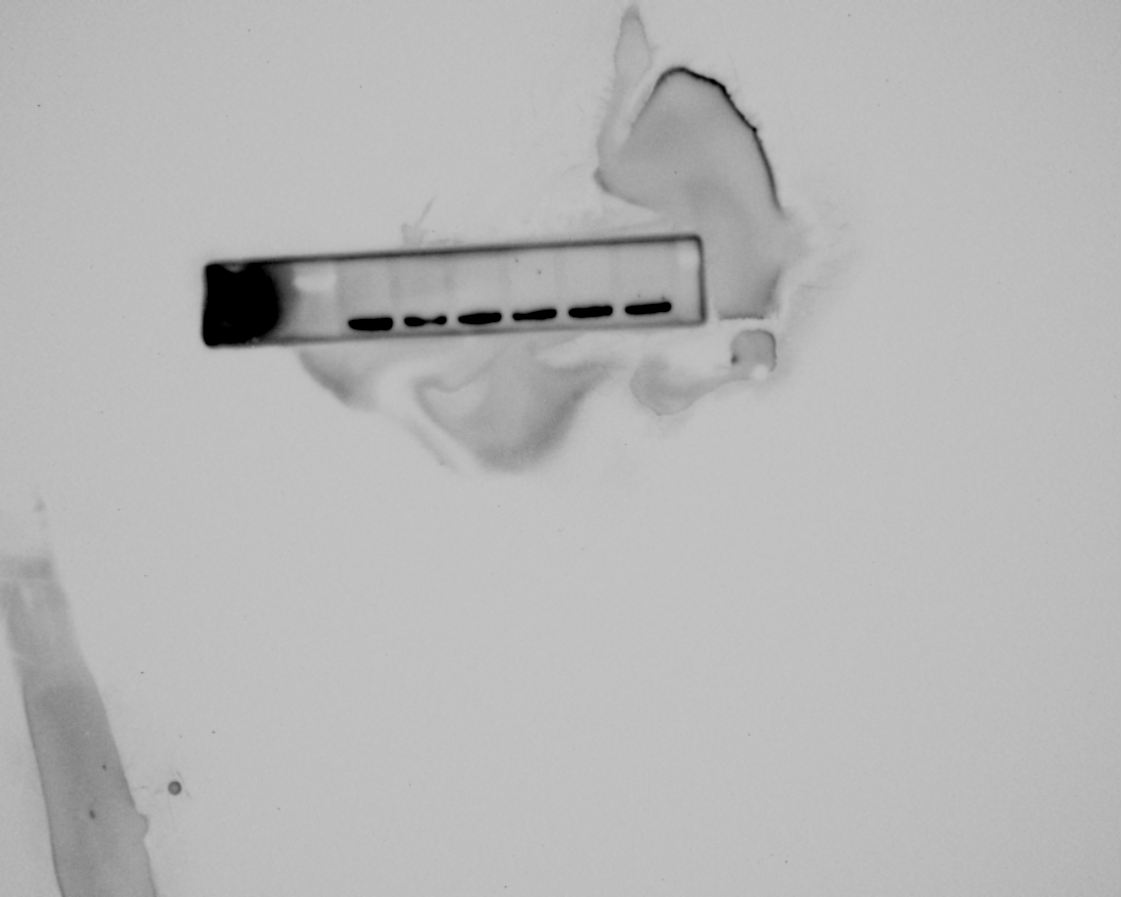

Supplement: Supplementary file 1 [file biology-12-00100-s001.zip › bcl-2.tif]

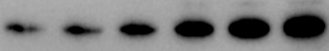

Supplement: Supplementary file 1 [file biology-12-00100-s001.zip › caspase3.tif]

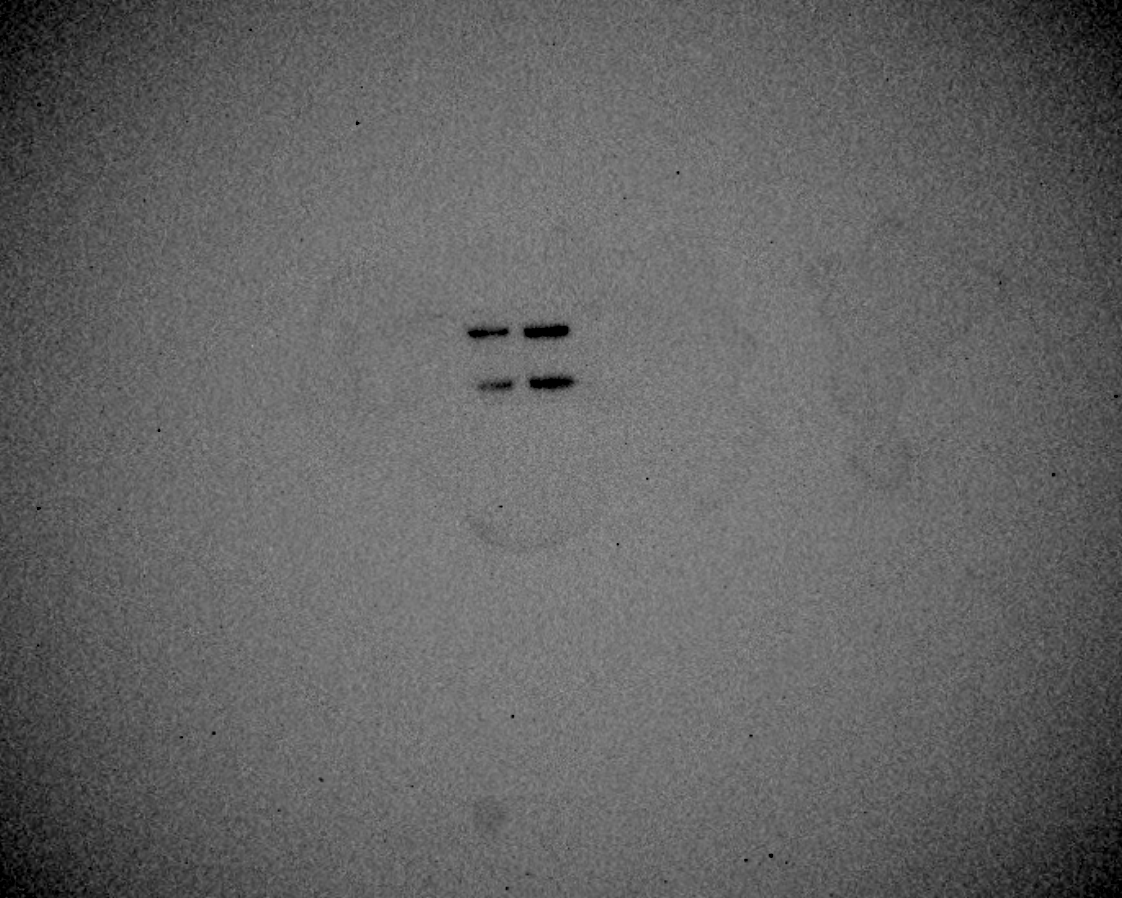

Supplement: Supplementary file 1 [file biology-12-00100-s001.zip › caspase9.tif]

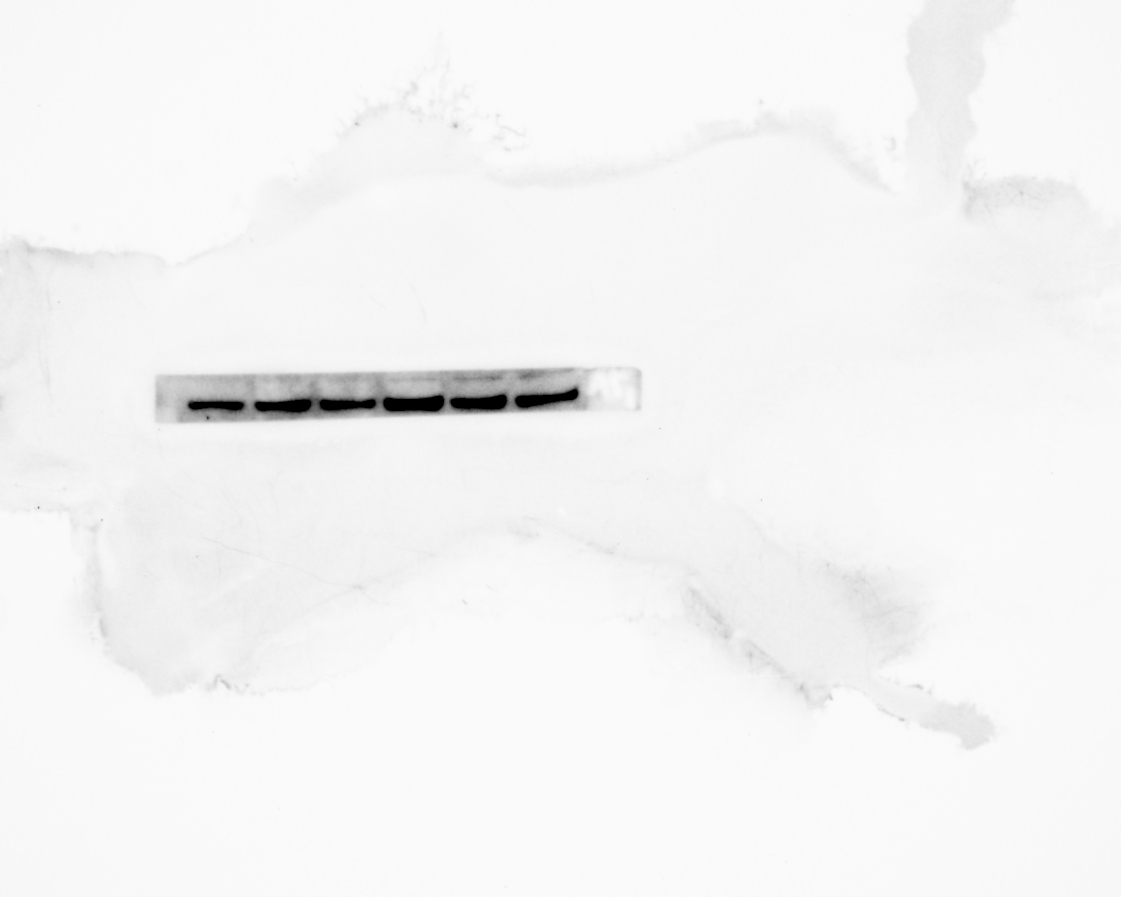

Supplement: Supplementary file 1 [file biology-12-00100-s001.zip › CAT.tif]

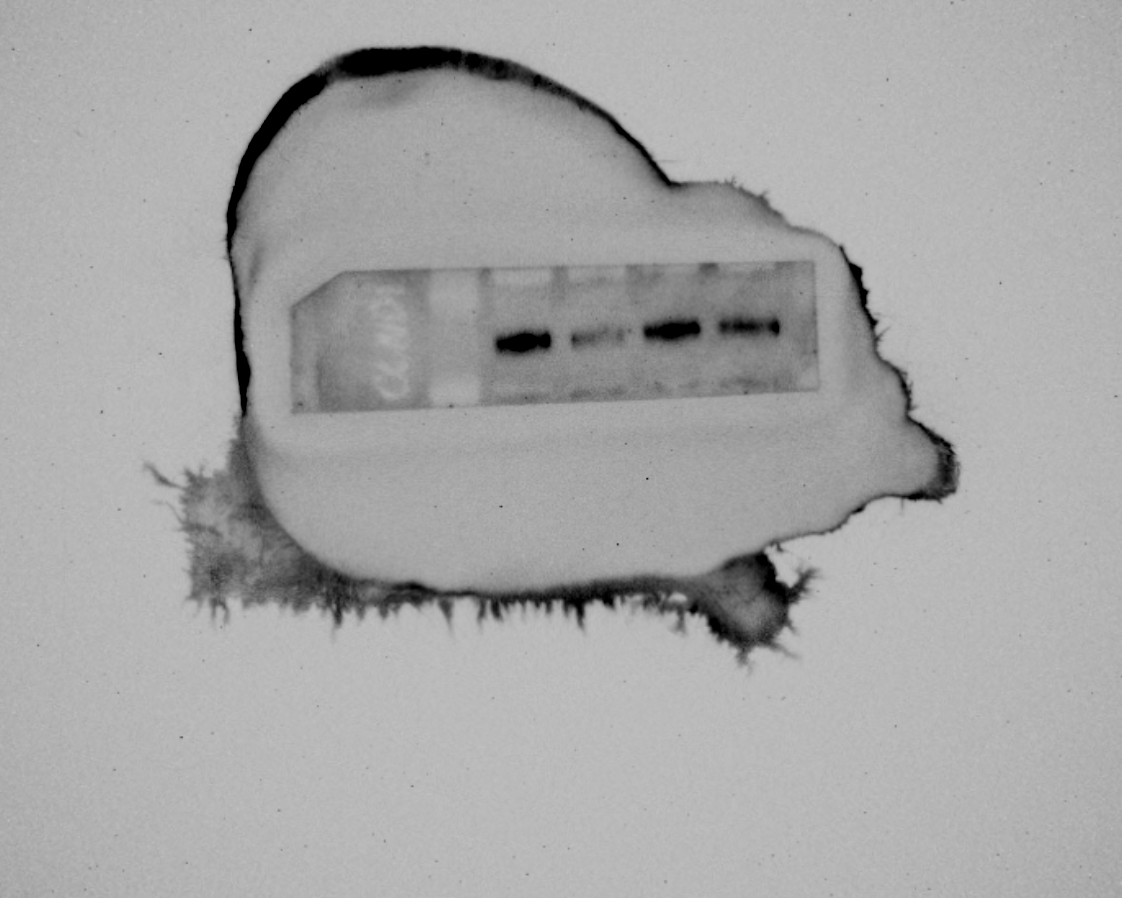

Supplement: Supplementary file 1 [file biology-12-00100-s001.zip › ccnd1.tif]

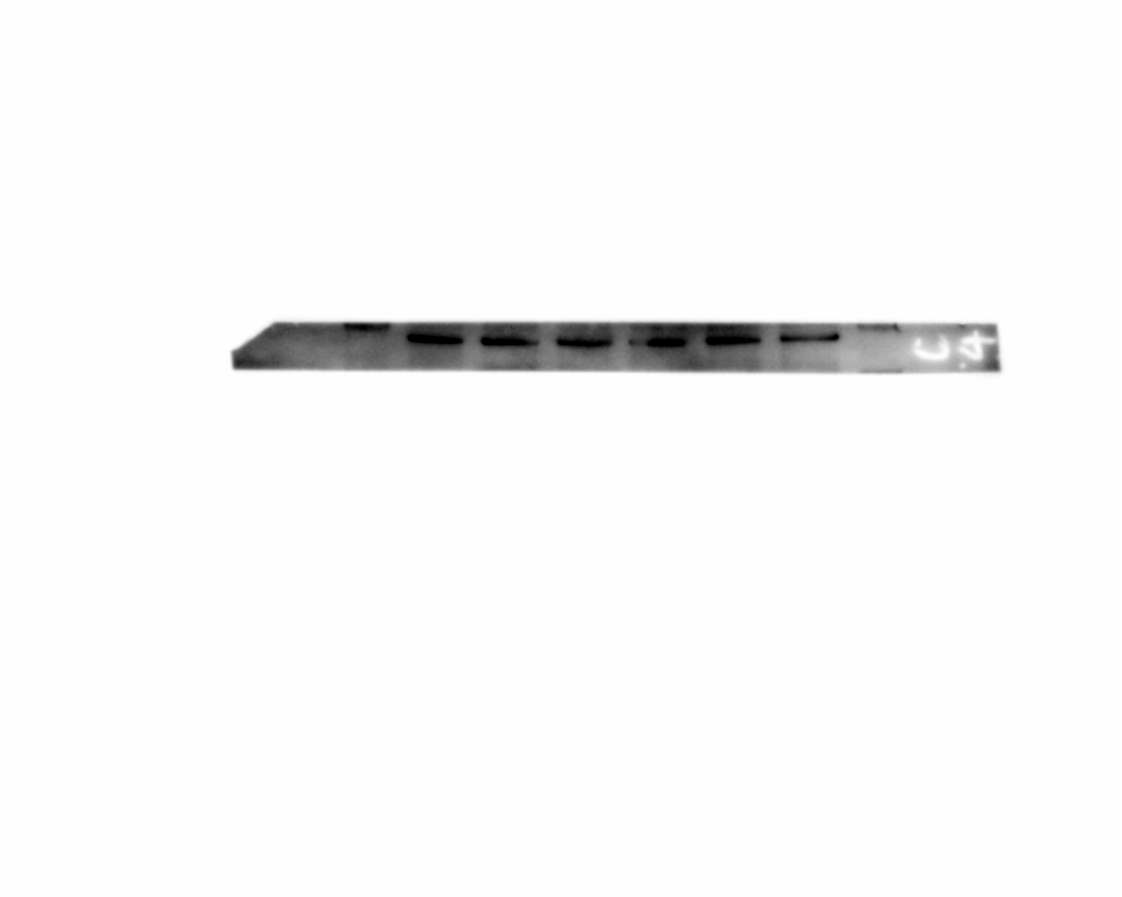

Supplement: Supplementary file 1 [file biology-12-00100-s001.zip › cdk4.tif]

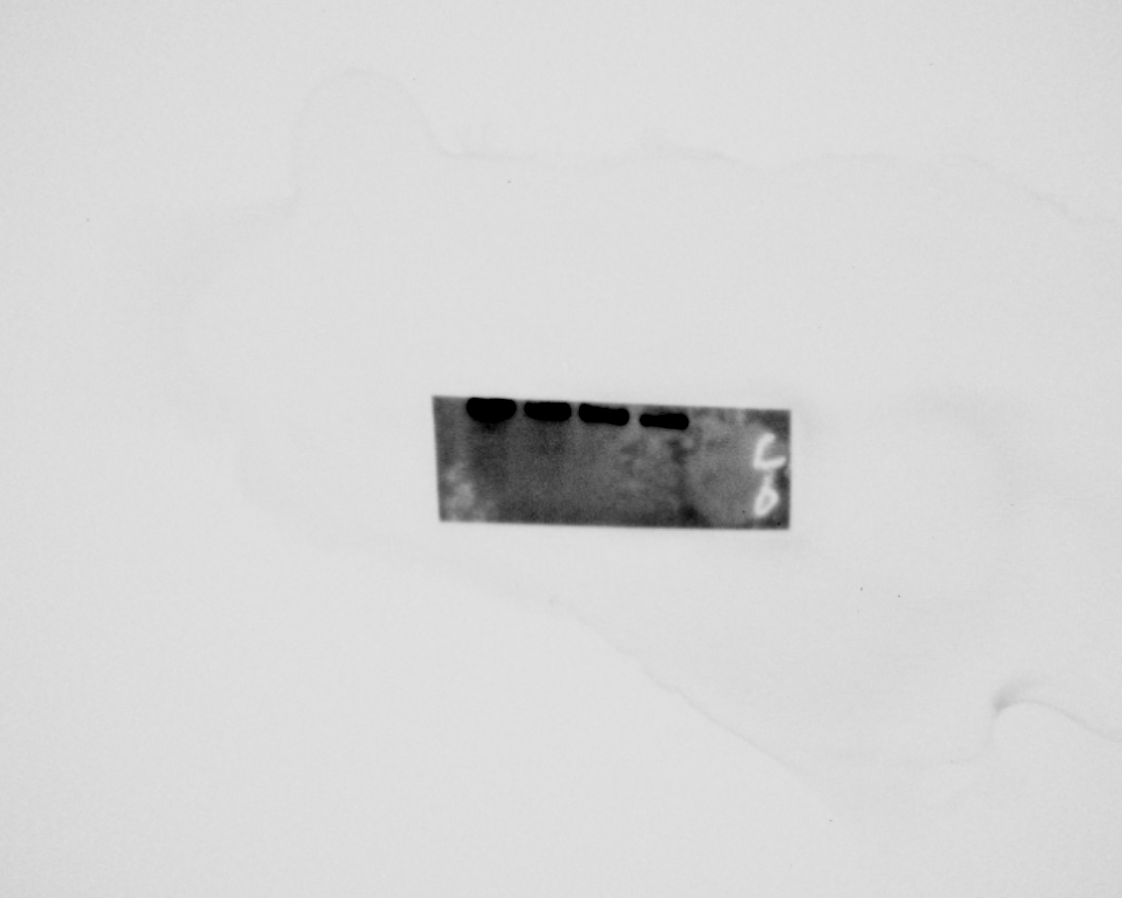

Supplement: Supplementary file 1 [file biology-12-00100-s001.zip › cdk6.jpg]

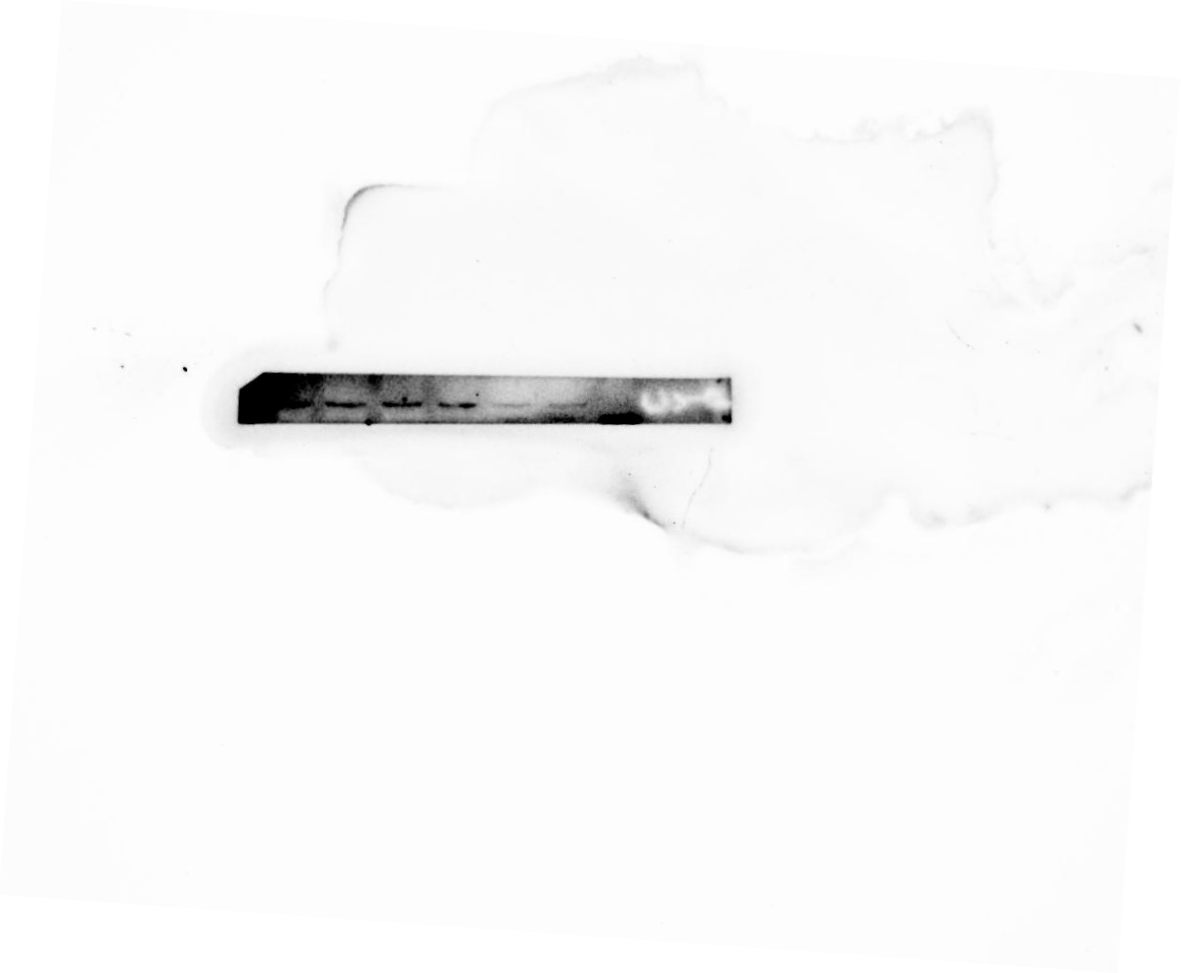

Supplement: Supplementary file 1 [file biology-12-00100-s001.zip › cyp11a1.tif]

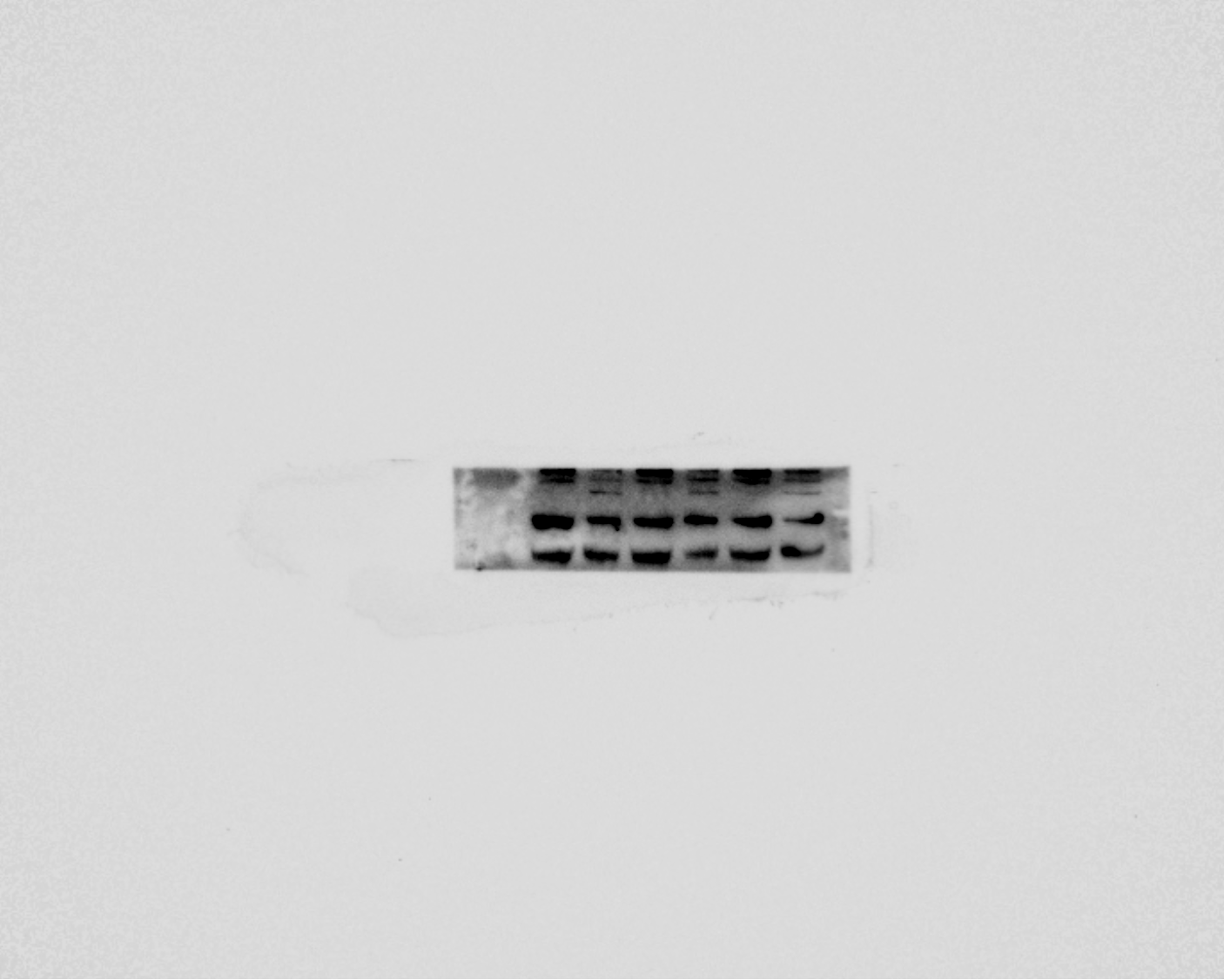

Supplement: Supplementary file 1 [file biology-12-00100-s001.zip › cyp19a1.tif]

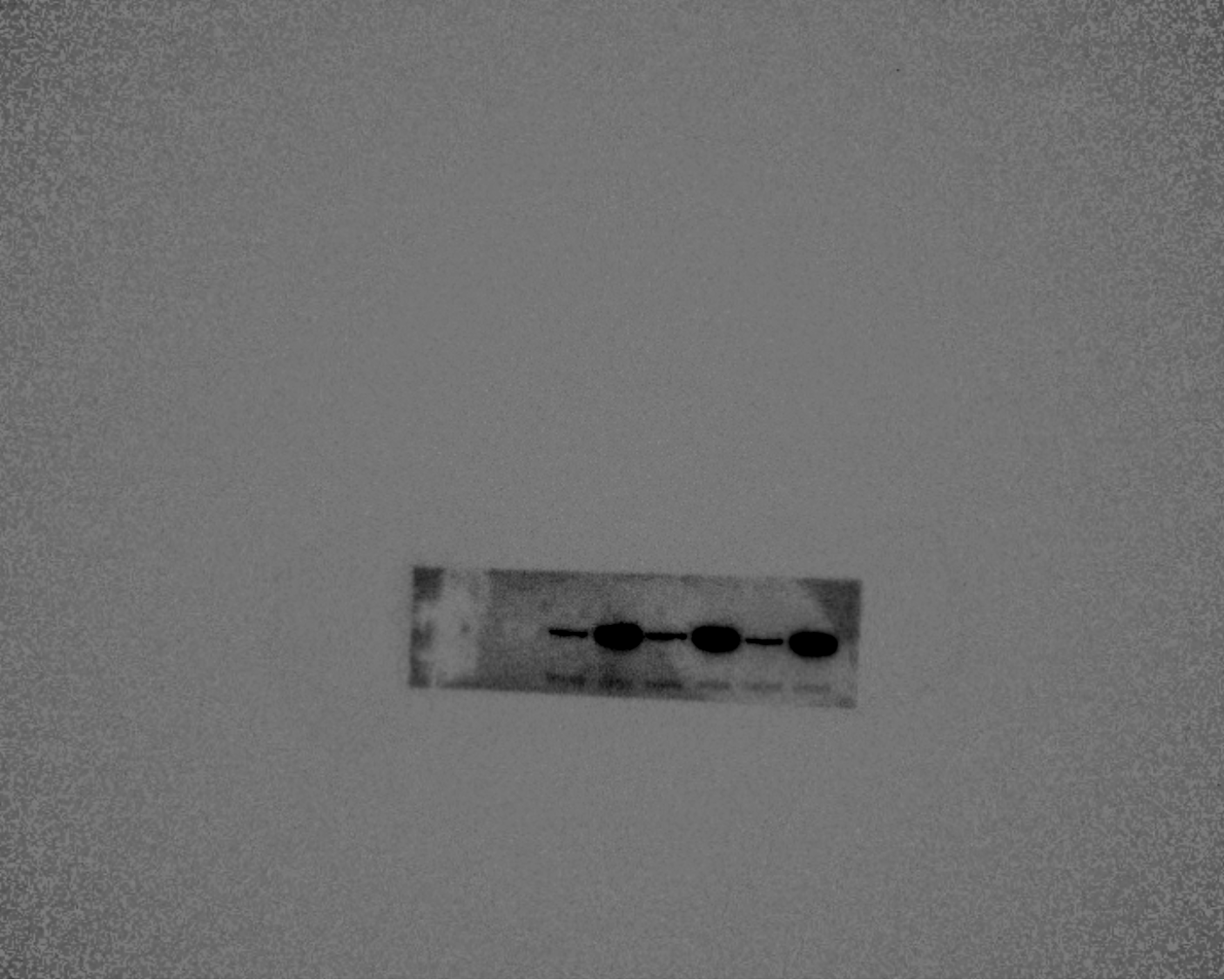

Supplement: Supplementary file 1 [file biology-12-00100-s001.zip › hsp70.tif]

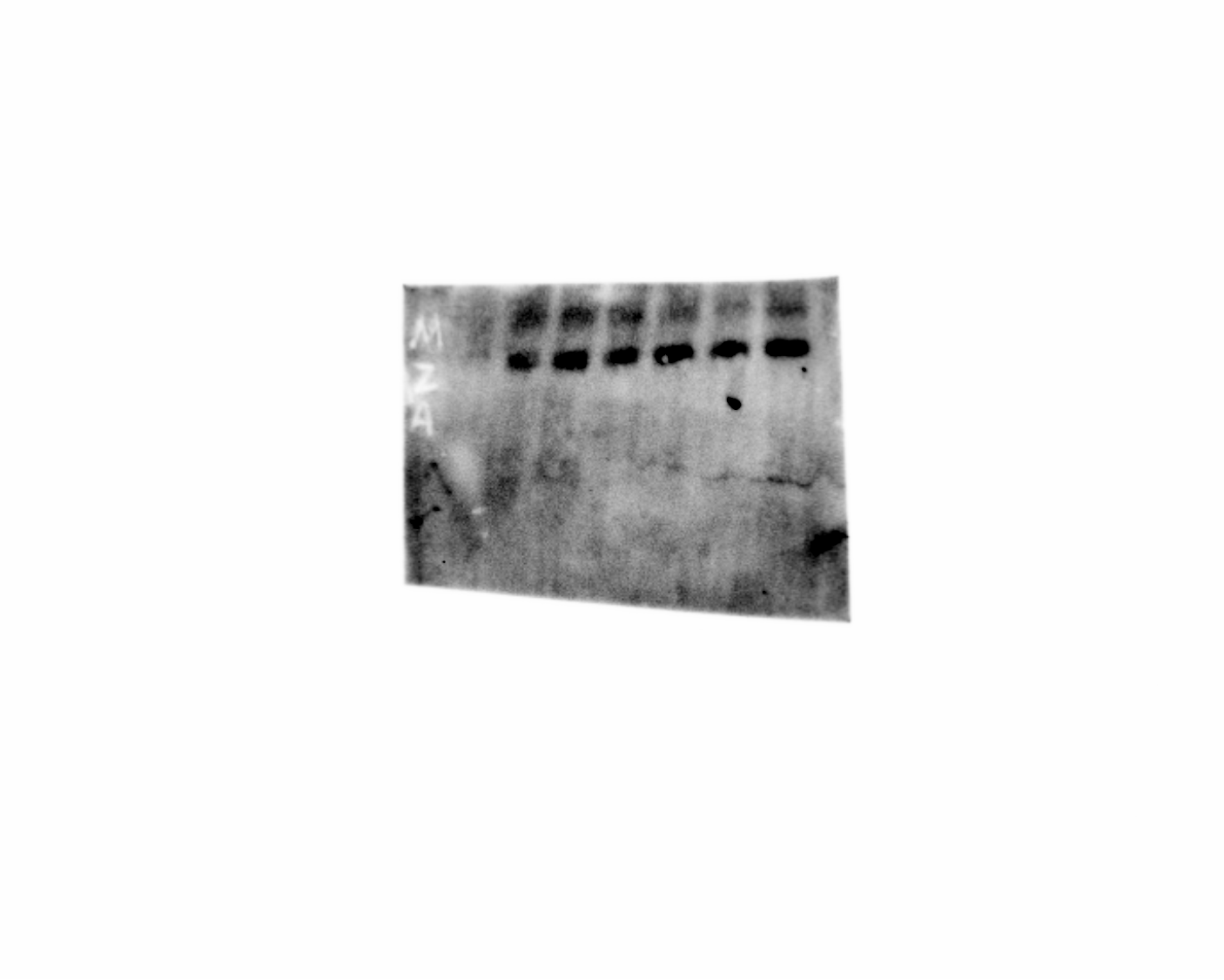

Supplement: Supplementary file 1 [file biology-12-00100-s001.zip › mt2a.tif]

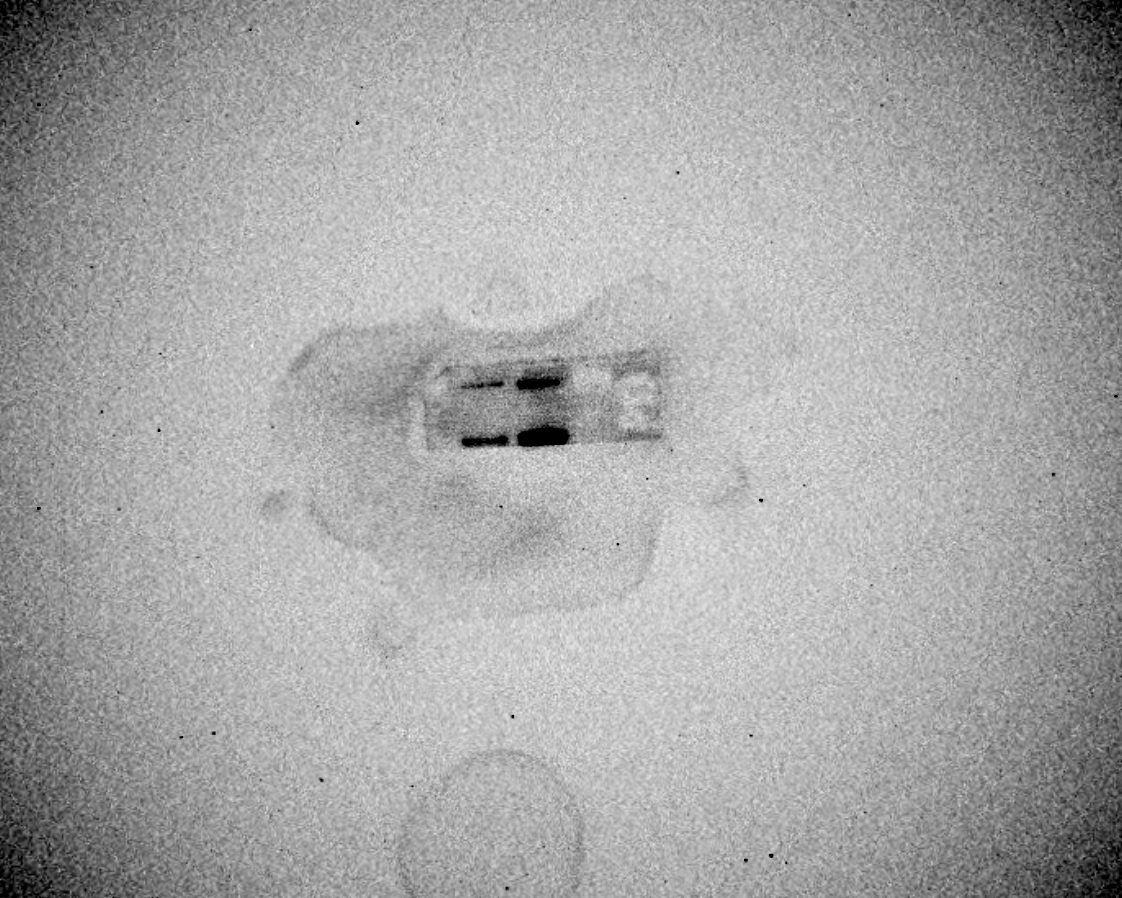

Supplement: Supplementary file 1 [file biology-12-00100-s001.zip › p53.tif]

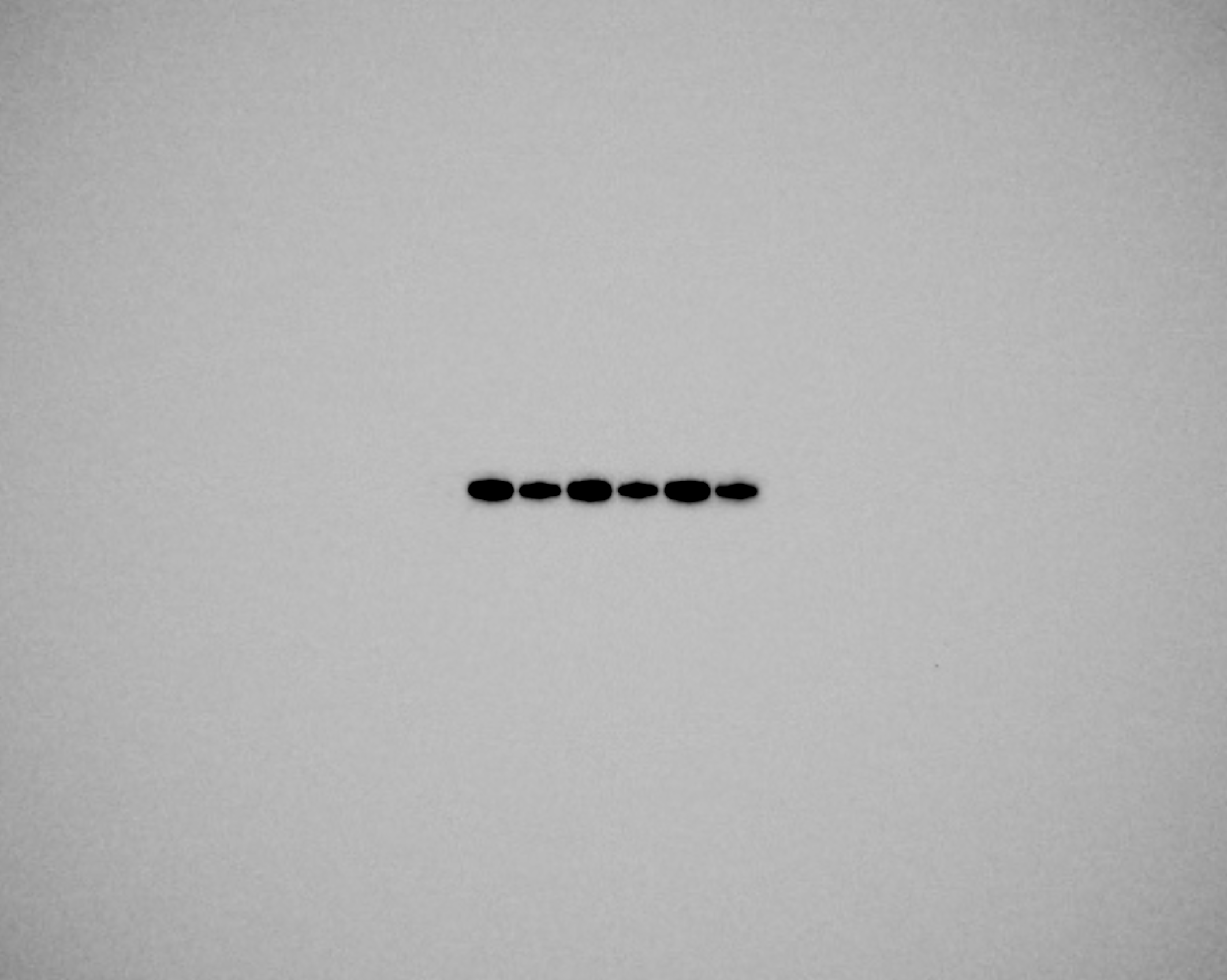

Supplement: Supplementary file 1 [file biology-12-00100-s001.zip › pcna.tif]

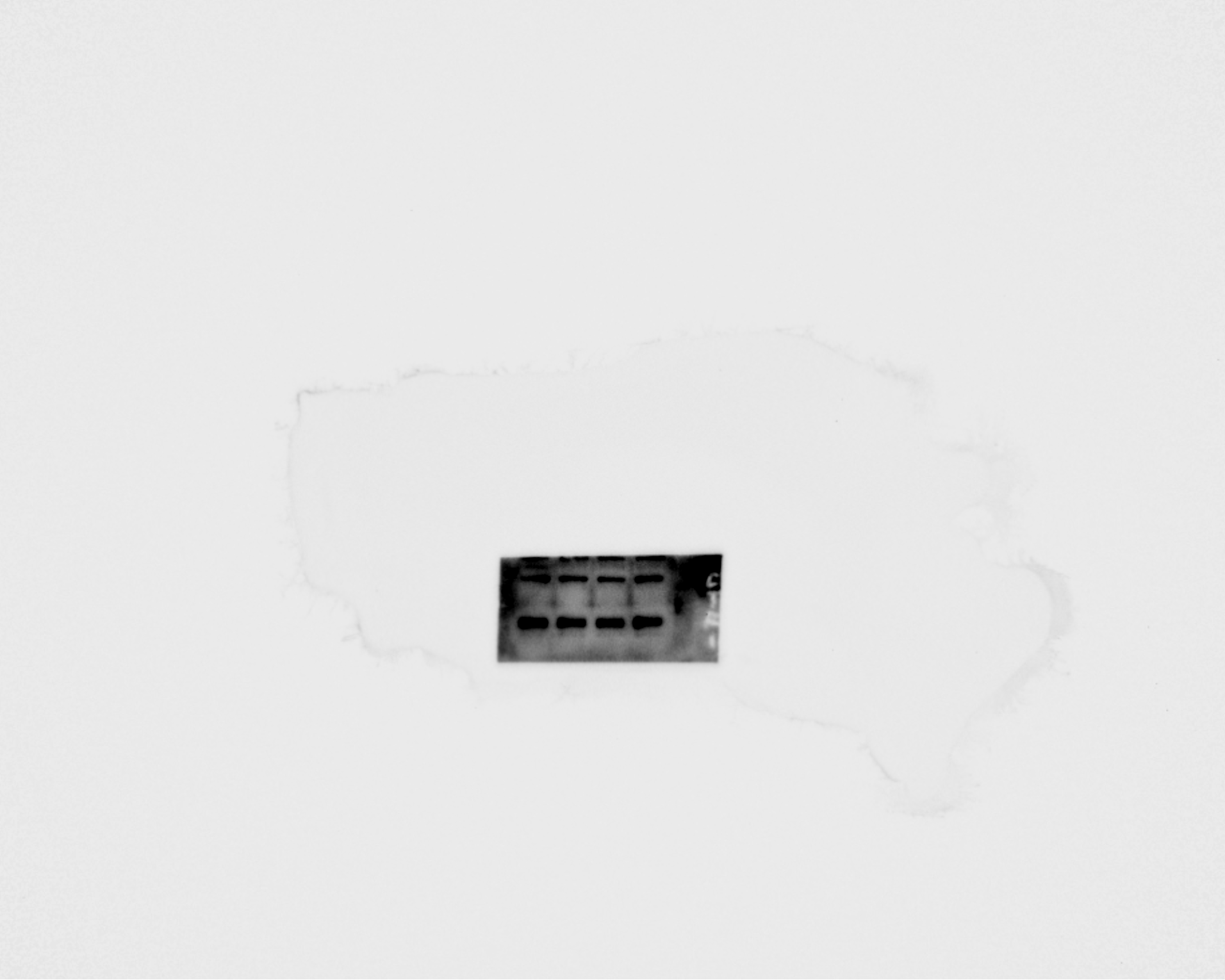

Supplement: Supplementary file 1 [file biology-12-00100-s001.zip › slc31a1.tif]

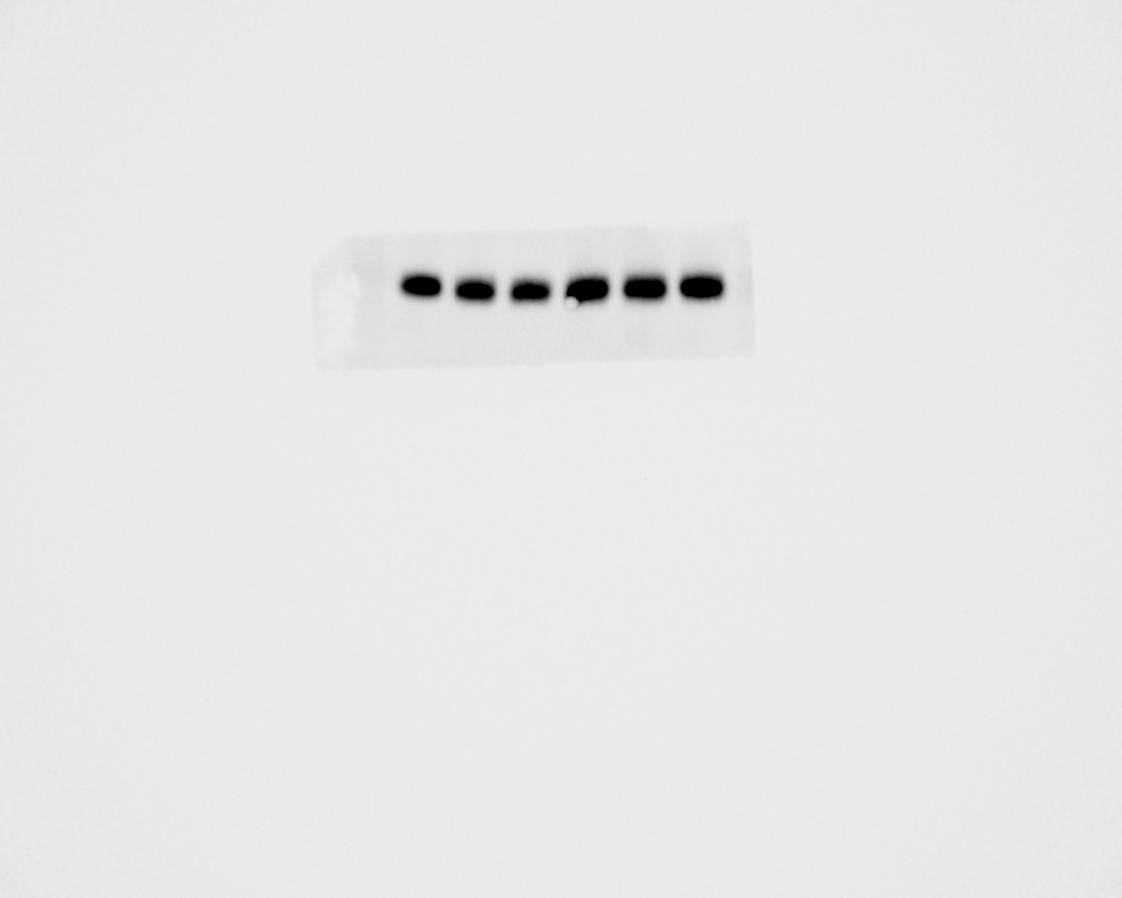

Supplement: Supplementary file 1 [file biology-12-00100-s001.zip › sod2.tif]

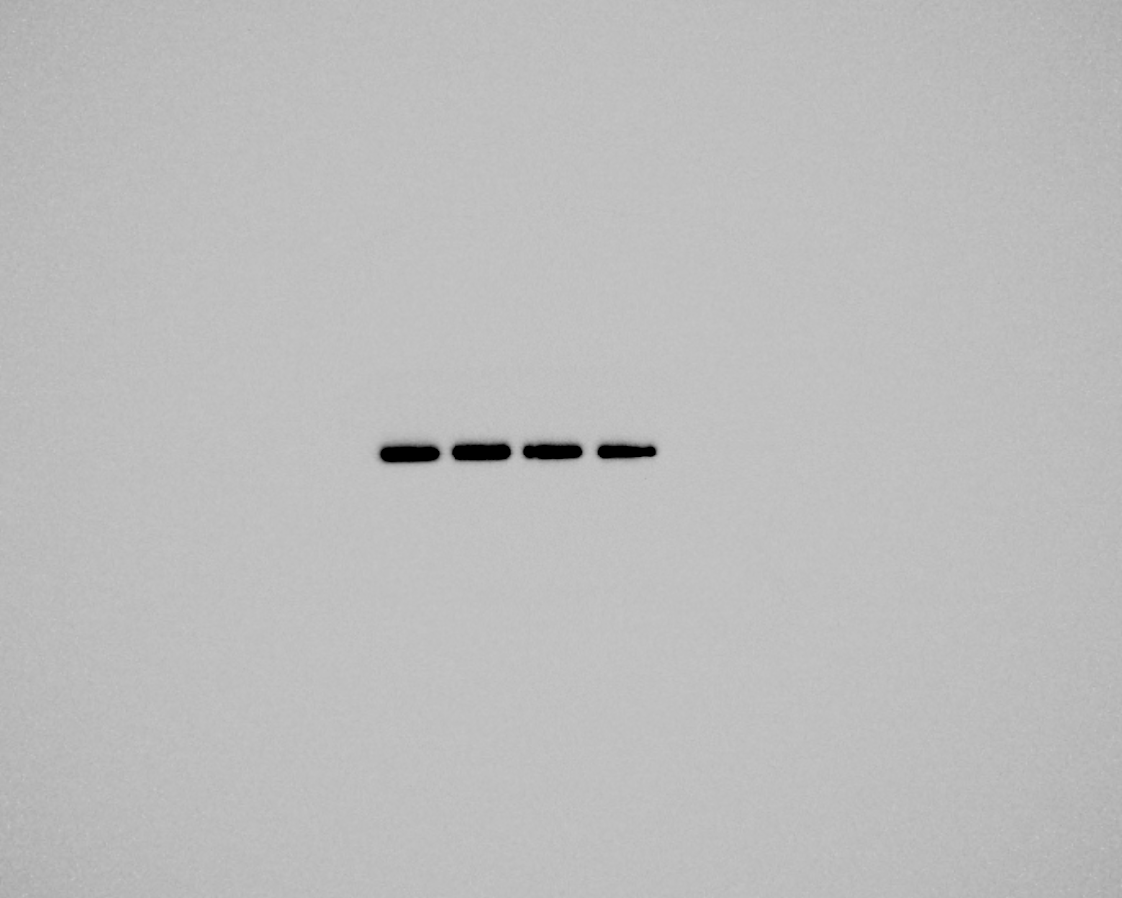

Supplement: Supplementary file 1 [file biology-12-00100-s001.zip › tubulin.tif]
